# Human Germline CRISPR-Cas Modification: Toward a Regulatory Framework

**DOI:** 10.1080/15265161.2015.1104160

**Published:** 2015-12-02

**Authors:** Niklaus H. Evitt, Shamik Mascharak, Russ B. Altman

**Affiliations:** ^a^ Stanford University

**Keywords:** clinical ethics, CRISPR-Cas, gene editing, gene therapy, genetic disease, germline modification

## Abstract

CRISPR germline editing therapies (CGETs) hold unprecedented potential to eradicate hereditary disorders. However, the prospect of altering the human germline has sparked a debate over the safety, efficacy, and morality of CGETs, triggering a funding moratorium by the NIH. There is an urgent need for practical paths for the evaluation of these capabilities. We propose a model regulatory framework for CGET research, clinical development, and distribution. Our model takes advantage of existing legal and regulatory institutions but adds elevated scrutiny at each stage of CGET development to accommodate the unique technical and ethical challenges posed by germline editing.

## CRISPR-Cas GERMLINE EDITING RAISES ETHICAL DILEMMAS

CRISPR-Cas (clustered regularly interspaced short palindromic repeats CRISPR-associated protein) systems form part of a bacterial adaptive immune response that recognizes and removes foreign DNA from the bacterial genome (Hsu, Lander, and Zhang 2014). This core ability to alter DNA has recently been generalized to enable specific addition and deletion of genomic DNA sequences as large as an entire gene and as small as a single nucleotide. Lower cost and smaller CRISPR-Cas components permit even broader editing of genomes (Ledford 2015; Ran et al. 2013). In the last two and a half years, researchers edited target genes in human somatic cells and human germline cells (Liang et al. 2015; Mali et al. 2013; Ran et al. 2013) and used CRISPR-Cas to eliminate disease in animal model systems (Wu et al. 2015). This unprecedented advance in germline engineering holds great promise for next-generation therapeutics but has sparked an ethical debate.

These developments in CRISPR-Cas genome engineering suggest that human germline editing trials may soon be pursued—raising challenges all along the spectrum of research to implementation. Baltimore and colleagues and Lanphier and colleagues raised ethical and safety concerns associated with CRISPR-Cas therapeutics—specifically the potential for exploitation in nontherapeutic uses, off-target genome modifications, and the existence of in vitro embryonic screening as a viable alternative (Baltimore et al. 2015; Lanphier et al. 2015). Webber noted an emerging legal debate surrounding the patentability of CRISPR-Cas modified genes (Webber 2014). Finally, Pollack and Lander examined the clinical ethics of this technology and cited concerns over the safety, cost–benefit analysis, possible applications in eugenics, and moral grayness inherent to genetic modification of human life (Lander 2015; Pollack 2015). Here, we offer a regulatory framework for human germline modification. We seek concrete policies that responsibly phase in therapeutic uses of CRISPR-Cas genome editing at a pace amenable to ethical inquiry.

## REGULATION IS THE MOST FEASIBLE PATH FORWARD

Rapid proliferation of CRISPR-Cas germline editing therapies (CGETs) for genetic disorders would entail nontrivial risk. It is critical that these technologies are adopted only after appropriately paced review, since improper dual use of germline modification would generate public mistrust of scientists seeking to develop CGETs.

International ban, temporary moratorium, regulation, and laissez-faire offer four approaches to CGET oversight (Bosley et al. 2015). A complete ban or temporary moratorium will be nearly impossible to enforce due to the low cost of CRISPR and heterogeneity of regional ethical codes. On the other hand, a laissez-faire approach creates risk that research will be conducted before ethical due diligence. Thus, regulation appears to be the most feasible policy. Fortunately, many of the institutions needed to regulate CGETs exist, although specific frameworks for oversight still need to be developed.

## RESEARCH, CLINICAL DEVELOPMENT, AND DISTRIBUTION OFFER OPPORTUNITIES FOR OVERSIGHT

CGET development can be divided into three phases: preclinical research, clinical trials, and postapproval distribution. Before each of these phases, financial and regulatory checkpoints should ensure that the proposed therapies meet safety and ethical guidelines. Prior to beginning research on any therapy, scientists must obtain funding and appropriate institutional ethics approval. In the transition from research to clinic, drug commercialization (e.g., the U.S. Food & Drug Administration [FDA]) and clinical practice (e.g., European Medicines Agency [EMA]) regulators ensure that product quality is sufficient for human trials through investigational new drug (IND) and clinical trial applications (CTA). At the juncture between clinical trials and commercialization, regulatory organizations must authorize marketing of a therapy and payers must reimburse it in order for the treatment to become widely available to the public.

### Guidelines for Research

From the start, CGETs must be vetted by funding bodies and institutional regulators for safety and benefit to patients before research can proceed. Preliminary safety standards for research should verify the specificity of CRISPR-induced modifications in a model cell line and ensure that new DNA introduced into the genome propagates through generations at a normal rate. One of the greatest risks of CGETs is the introduction of alleles with unintended side effects that are only recognized generations after initial gene editing (Baltimore et al. 2015; Lander 2015; Lanphier et al. 2015). Currently, a harmful gene edit cannot be removed from the population once introduced into the germline. Until we develop the technology to remove deleterious edits, we should not accelerate the pace at which edits can spread. It follows that the use of gene drives in conjunction with germline CRISPR should be prohibited in any project that lacks a validated reversal strategy (see further discussion). The benefit of a therapeutic gene edit with guaranteed transmission does not outweigh the cost of its unforeseen complications. If a gene edit is beneficial, recipients will likely choose to provide the same treatment to their children. If not, an irreversible modification should not spread through the population in an accelerated manner.

Indeed, it may be possible to remove germline edits from the population through a modification of the gene drive overwrite strategy proposed by Church (Esvelt et al. 2014). We envision transduction of embryos with secondary gene programs that are chemically induced and that precisely reverse the original therapeutic edit (Mezhir et al. 2005). Any CGET should include a companion reversal mechanism. Furthermore, chemical induction of reversal mechanisms must be orthogonal to natural biochemistry so that removal of original gene edits is not accidentally triggered. Obviously, significant research is needed before such reversal mechanisms are made a reality.

In addition to being safe, germline modifications should offer clear benefits to patients (Lander 2015). We hold that two cases exist for appropriate research of CGETs. In the first case, therapeutic effects made possible by germline editing cannot be achieved by embryo selection and prenatal genetic diagnosis (PGD). In monogenic diseases, CGETs confer unique therapeutic benefits when one parent is homozygous for a dominant disease state or both parents are homozygous for a recessive disease state (Lander 2015). Polygenic diseases are often too complex to remedy without risking harmful side effects (Lander 2015). In the second case, diseases with a large potential patient population present an ethical use of germline editing, even if embryo selection via PGD could be used by parents to avoid having a child with disease. We hold that embryos bear an intermediate moral status between nonhuman life and a fetus. As such, embryonic destruction over the course of a research program should be minimized. However, disease-state embryos will also be destroyed every time parents conduct PGD during an in vitro fertilization (IVF) cycle. As a result, developing a CGET is morally justified when the population-wide embryo loss in PGD will likely surpass the embryo loss during CRISPR research.

If a proposed therapy passes either of the preceding ethical tests, oversight committees should ask investigators to demonstrate proof of concept by applying putative therapeutic gene edits to relevant somatic cells and multigenerational animal models. Somatic proof-of-concept trials provide a platform to refine the phenotype (i.e., penetrance, expressivity) of CGETs in the eventual target cell type. These trials also lower the ethical burden of germline experiments by minimizing embryo destruction. Diseases specific to the germline would bypass this set of trials. However, many diseases are tissue localized. For these diseases, preclinical somatic cell proof-of-concept trials are an important means of diminishing the ethical hazards associated with developing CGETs. Given the risk of side effects in future generations, a CGET should also be validated in multigenerational animal models of increasing complexity (e.g., rat, pig, dog) before consideration for human clinical trials.

Normally, an institutional review board (IRB) would be responsible for regulating early-stage human therapeutics research. Since embryos are not considered human subjects and are not afforded the same protections outlined by the Belmont Report (e.g., autonomy, informed consent), CGET research does not fall clearly under the purview of an IRB. Furthermore, IRBs are prohibited from considering long-term social ramifications when deciding to approve research. Since CGETs could have a lasting impact on the human gene pool, it would be wise to delegate oversight of this technology to specialized committees composed of diverse stakeholders. Local oversight committees should be composed of researchers, physicians, ethicists, and community members with nonconflicting interests, much like stem cell research oversight (SCRO) committees. Guidelines for the membership and powers of these committees should be standardized by federal mandate to ensure consistent policy at a national level. Furthermore, just as SCRO committees are distinguished from IRBs by their broader focus, CGET committee responsibilities should extend beyond human subject protection.

Currently, the National Institutes of Health (NIH) refuses to fund proposals for CGETs (Collins 2015). We support this moratorium on funding while the biotechnology community refines an appropriate ethical and regulatory framework. Once a robust framework is in place, we believe that the NIH should lift funding restrictions and accept conservative CGET research proposals as long as they conform to the rigorous guidelines above. When (1) sufficiently nuanced in vitro human tissue models are developed to run somatic cell proof-of-concept trials and (2) the effects of increasingly complex genome edits are better understood in animal models, we recommend expanding the scope of CGETs approved for embryonic research.

### Guidelines for Clinical Development

After a CGET has been funded and validated in the laboratory, the FDA must approve the therapy before clinical use. Prior FDA policies concerning gene transfer therapies readily port over to CGETs. The FDA defines gene transfer technology as “any exposure to gene therapy products … by any route of administration” and gene therapy products as “all products that mediate their effects by transcription and/or translation of transferred genetic material and/or by integrating into the host genome and that are administered as nucleic acids, viruses, or genetically engineered microorganisms” (FDA 2006, 2–4). CRISPR-Cas systems certainly fall under this broad purview, as they are virally delivered, genomically stored, and mediate their effects via transcriptional machinery.

Before Phase I clinical trials, care should be taken to receive parental informed consent. One could argue that germline modification would lead to “generations of nonconsent,” as genome edits are propagated first to a child and then to subsequent generations without their express permission. We hold that the child's informed consent is not warranted for the first edited generation, as parents are frequently permitted to make medical decisions on behalf of their children. Informed consent from subsequent generations is still not warranted, as nonexistent beings cannot be presumed to have that right (Munson and Davis 1992). As such, parental informed consent should be sufficient for participation in CGET clinical trials.

However, obtaining completely informed consent is made problematic by the possibility of unanticipated latent side effects. Current FDA standards mandate that “advised sponsors [observe] subjects for potential gene therapy-related delayed adverse events for a 15 year period,” but do allow for shorter observation times if the “risk of delayed adverse events is low” (FDA 2006, 6). It is reasonable to assume that this risk is comparable when somatic cells are modified by traditional gene transfer or more targeted CRISPR-Cas strategies; however, the fact that germline genome modifications could be risky over the entire lifetime of an organism and all its future progeny necessitates a higher standard of informed consent for entry into clinical trials.

Phase I–III clinical trials should be carefully scoped to make CGETs accessible to patients as soon as possible while conclusively demonstrating safety and efficacy. We agree in spirit with Lanphier et al. that “philosophically or ethically justifiable applications for this technology … are moot until it becomes possible to demonstrate safe outcomes and obtain reproducible data over multiple generations” (Lanphier et al. 2015). However, multigenerational Phase I–III trials may be impractical. Mandatory multigenerational Phase IV trials could still confirm positive long-term outcomes while mitigating excessive time burdens during pre-market development.

### Guidelines for Distribution

After FDA approval and commercialization, CGETs may be distributed via IVF clinics. Insurance companies are currently not obligated to cover IVF-related expenses, but they may seek to provide lower insurance deductibles to individuals who received germline editing. This would disadvantage those who choose to conceive naturally or were born before the advent of germline engineering, while creating a financial “gene drive” promoting the widespread adoption of the therapeutic. Clearly, access to efficacious germline editing could also be problematic for prospective parents of lower socioeconomic status, who do not possess the financial means required for IVF. Buyer-side legislation should be enacted, or best practices should be adopted, to ensure justice for those who cannot or choose not to use this technology. Legislation must also respect patient autonomy. For example, insurance companies should not be permitted to raise deductibles of deaf parents who choose to conceive a deaf child; regardless of the morality of this decision, it is still legally viewed as a matter of parental autonomy (Zimmerman 2009).

## AN INTEGRATED FRAMEWORK FOR CGET REGULATION

**Figure 1.**
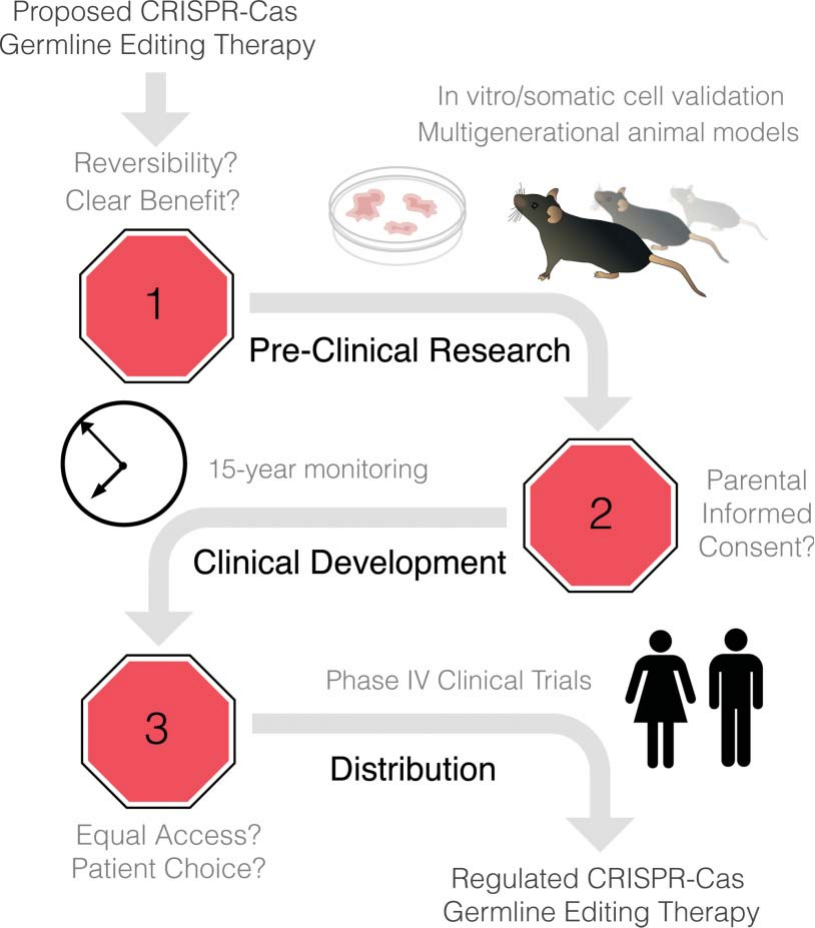
A model regulatory pathway for developing CRISPR germline editing therapies.

We have proposed a model regulatory framework for CGETs (Figure 1) to meet the technical and ethical demands specific to these therapies. Together, our recommendations (see the following) address underdeveloped safety mechanisms, increased risk of multigenerational side effects, ethical complications of medical research and practice involving human embryos, and concerns about equal access to CGETs.\raster="rgFigUAJB_A_1104160_F0001_B"

### Prior to Preclinical Research

Grant agencies and CGET-specific oversight committees verify that:

- Specific CGET-reversal mechanisms orthogonal to natural biochemistry have been designed.
- Proposed CGETs contain no gene drives.
- CGETs address monogenic diseases with no alternative treatment or widespread diseases for which embryo loss through PGD treatment will dwarf embryo loss during CGET research.

### During Preclinical Research

Researchers show proof-of-concept technical feasibility, safety, and efficacy in:

- Somatic cell models of the targeted tissue type.
- Multigenerational animal models of increasing complexity.

### Prior to Clinical Development

CGET-specific oversight committees and federal regulators ensure that:

- CGET clinical trial protocols include elevated standards of parental informed consent for participation.

### During Clinical Development and Distribution

CGET developers conduct:

- Fifteen-year Phase I–III clinical trials in accordance with gene transfer therapy standards prior to BLA submission.
- Mandatory multigenerational Phase IV postmarketing surveillance trials.

### Prior to Distribution

Federal legislators pass legal safeguards that:

- Preserve the autonomy of and combat discrimination against patient groups who choose to opt out from CGETs.
- Ensure equal access to CGETs for marginalized patients who opt in (e.g., low socioeconomic status patients).

## FROM FRAMEWORK TO POLICY

Although there has been much recent conversation about the ethical dilemmas presented by CRISPR-Cas germline editing therapies (CGETs), there has been little discussion of how such therapies would be responsibly developed in practice. Clearly, the unprecedented promise and peril of CGETs warrant careful consideration. It should be noted that many existing regulatory frameworks could already accommodate CGETs with elevated scrutiny at each step. While we have presented a more stringent model for regulation demanded by CGETs, this discussion opens the door to an essential debate that must occur before the community continues research on these potentially powerful therapies.
